# Shaping the future of migraine targeting Calcitonin-Gene-Related-Peptide with the Disease-Modifying Migraine Drugs (DMMDs)

**DOI:** 10.1186/s10194-019-1009-9

**Published:** 2019-05-23

**Authors:** Paolo Martelletti, Lars Edvinsson, Messoud Ashina

**Affiliations:** 1 Department of Clinical and Molecular Medicine, Sapienza University, Rome, Italy; 2 Department of Clinical Sciences, Lund University, Lund, Sweden; 3 Department of Neurology and Danish Headache Center, University of Copenhagen, Copenhagen, Denmark

**Fig. 1:**
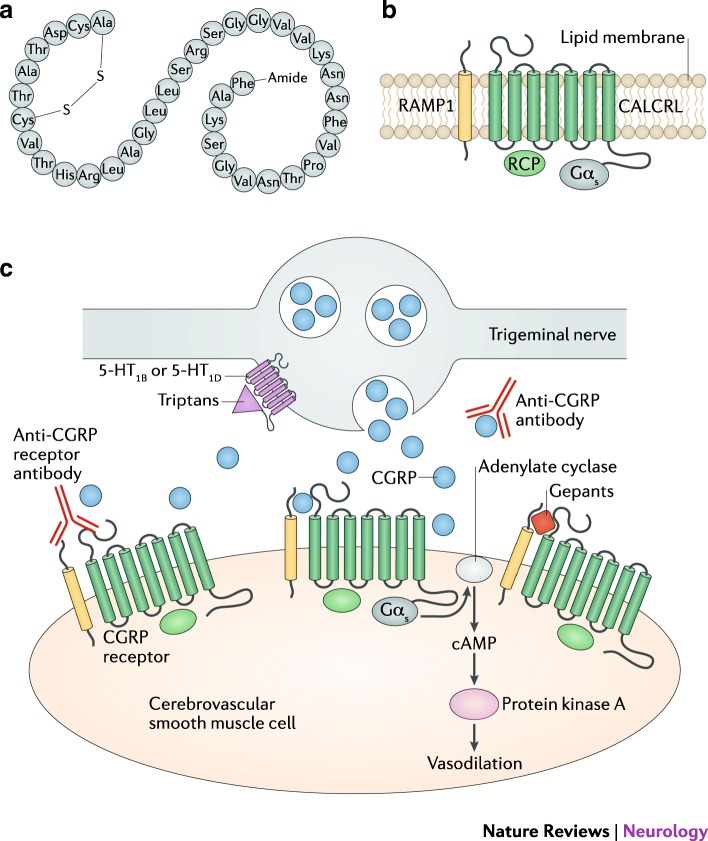
The overall picture on how the new era of CGRP pathway interacting molecules is given below. These drugs are designer molecules that have been constructed as anti-migraine [1]

Speaking of a new pharmacological class, which includes the monoclonal antibodies for Calcitonin Gene Related Peptide or its receptor (CGRP (r)) and gepants seems to be a simple thing, given the enormous expectation that has arisen around them and the enormity of data that we have seen. The numerous randomized control trials (RCTs) of the four siblings, erenumab, fremanezumab, galcanezumab, eptinezumab, show strong evidence for their differentiated use in episodic, chronic and refractory migraine, as second-line drugs for now [1, 2]. Even studies on gepants are moving, albeit with differentiated speed, in the area of ​​prevention or acute treatment of migraine. We have on the horizon the reappearance of a pharmacological class composed of atogepant, rimegepant and ubrogepant [3] (Fig. 1).

We now have recommendations from the European Headache Federation on the use of monoclonal antibodies that will act as a guide and as a beacon in the coming years to be regularly updated as new scientific evidences will be available [4].

The CGRP(r) target in migraine has solid retrospective roots, moving its first steps in the world of the trigeminovascular system of CGRP over 30 years ago, when it was demonstrated that the perivascular administration of the powerful vasoconstrictor, norepinephrine, produced the spontaneous counteract dilation response of CGRP [5, 6]. This was coined trigeminovascular reflex, aborted by lesions of the trigeminal nerve.

Subsequent work revealed that CGRP was the neuronal responsible messenger [7].

The journey then had several stages, and the caravan was always enriched with new data, up to the current stage we celebrate today, a new pharmacological target dedicated to migraine and based on monoclonal antibodies directed against either CGRP or the CGRP receptor or on small molecules with antagonism activity towards the CGRP [1, 8].

Interfering with the important release of CGRP at the level of neurons and fibers of the Trigeminal Ganglion (TG), activated by the still unknown natural switch-on originating putatively in the Central Nervous System (migraine generator), CGRP (r) Mabs are the most recent drug barrier able to tackle migraine pain onset [1, 2].

Translational medicine has then transferred this basic science research data to the human science, setting-up an experimental human migraine model leading to novel vascular mechanics insights as well as neuroimaging studies through the use of functional MRI (fMRI) blood oxygenation level dependent (BOLD) revealing that CGRP acts outside the Blood-Brain-Barrier (BBB) ​​ [9].

The necessity to have these drugs now available for patients opens a new phase, stimulates new studies and generates the expectation of real-world data. In the meantime, we must consider that the clinical application of these drugs directed to the CGRP, members of the first and only pharmacological classes created de novo for migraine treatment, acting on the CGRP biomarker of disease [10], maintain their long-term efficacy reverting this way the migraine disease to a *quasi*-silent state [11].

With these premises, we can define this CGRP new pharmacological class as Disease-Modifying-Migraine-Drugs (DMMDs), for the benefit of the huge number of patients needing therapies that, if promptly used, can slow down or freeze or revert the natural course of migraine [12].

Only in this way will we be able to respond to an ever more evident cultural movement that, based on scientific data, wants to categorize migraine not as an ineluctable family trace but as an endemic disease, which represents one of the major public health priorities worldwide [13, 14].
